# Altered Resting-State Functional Connectivity in Internet Gaming Disorder: Convergent Evidence and Independent Validation

**DOI:** 10.1016/j.abrep.2026.100703

**Published:** 2026-05-02

**Authors:** Jiawen Tian, Hui Zhang, Xinyu Wang, Hongyu Zhang, Longyao Ma, Bohui Mei, Mengzhe Zhang, Yan Lang, Yarui Wei, Shaoqiang Han, QingQing Lv, Yong Zhang

**Affiliations:** a Department of Magnetic Resonance Imaging, The First Affiliated Hospital of Zhengzhou University, Zhengzhou, China; b Zhengzhou Key Laboratory of Brain Function and Cognitive Magnetic Resonance Imaging, Zhengzhou, China; c the Third Affiliated Hospital of Zhengzhou University, Department of radiology, China; d Department of Psychiatry, The First Affiliated Hospital of Zhengzhou University, Zhengzhou, China

## Abstract

**Background:**

Internet gaming disorder (IGD) has attracted increasing attention worldwide. However, findings from resting-state functional connectivity (RSFC) studies have remained inconsistent, making it difficult to identify robust and reproducible neural alterations. To address this issue, we performed a coordinate-based synthesis of RSFC studies involving IGD-related conditions and examined whether the principal convergent findings could be replicated in an independent IGD cohort.

**Methods:**

Convergent RSFC abnormalities across eligible studies were identified using anisotropic effect size–signed differential mapping (AES-SDM). Clusters derived from the meta-analysis in the middle frontal gyrus and midline medial frontal/cingulate-related region were defined as regions of interest for independent validation. Resting-state fMRI data were collected from 58 individuals with IGD and 38 healthy controls, followed by seed-based RSFC analyses. Group differences were examined using two-sample t-tests with Gaussian random field correction, and brain–behavior associations were evaluated using Pearson correlations.

**Results:**

Seventeen studies met the inclusion criteria for meta-analysis. Convergent RSFC abnormalities were mainly observed in the median cingulate/paracingulate region and bilateral middle frontal gyrus. In the independent sample, individuals with IGD showed significant seed-based RSFC alterations involving both the middle frontal gyrus seed and the midline medial frontal/cingulate-related seed. Connectivity strength related to the middle frontal gyrus seed was positively associated with Internet Addiction Test scores.

**Conclusions:**

These findings provide convergent evidence for abnormal resting-state connectivity in IGD, particularly in frontal and midline regions involved in executive control and self-referential processing. Independent validation further supports the reproducibility and potential clinical relevance of these regionally specific alterations.

## Highlights

• Meta-analysis identified convergent fronto-midline RSFC abnormalities in IGD.
• Independent seed-based RSFC validated meta-analytic clusters in IGD.
• Middle frontal and medial frontal seeds showed increased RSFC in IGD.
• Middle frontal seed connectivity correlated with IAT severity in IGD.
• Findings support reproducible executive-control dysfunction in IGD.

## 1 Introduction

Internet gaming disorder (IGD) is recognized as a behavioral addiction marked by persistent and excessive gaming, reduced control over gaming behavior, and continued involvement despite negative consequences. As defined in the Diagnostic and Statistical Manual of Mental Disorders (5th ed.; DSM-5), IGD has drawn increasing attention because of its considerable psychological and social burden (American Psychiatric Association, 2013, Regier et al., 2013, Kuss and Lopez-Fernandez, 2016, Weinstein and Lejoyeux, 2010). Previous behavioral and neuroimaging studies suggest that IGD is associated with impaired executive control, heightened reward sensitivity, craving-related responses, and reduced self-regulatory capacity (Bechara, 2005, Zhou et al., 2012, Kuss and Lopez-Fernandez, 2016). Within the Interaction of Person-Affect-Cognition-Execution (I-PACE) framework, these features are understood as arising from interactions among individual predispositions, affective and cognitive responses, cue-reactivity and craving, and diminished executive control. An imbalance between reward-driven motivational processes and weakened top-down regulation may contribute to the persistence of maladaptive gaming behavior (American Psychiatric Association, 2013, Brand et al., 2016, Brand et al., 2019, Weinstein and Lejoyeux, 2020).

Resting-state functional magnetic resonance imaging (rs-fMRI) offers a useful approach for examining intrinsic functional connectivity and large-scale patterns of brain organization without the need for task performance (Canario et al., 2021, Sutherland et al., 2012). Among the neural systems involved in addictive behaviors, the central executive network (CEN) and the default mode network (DMN) are especially relevant, particularly in relation to addiction-related disturbances in control and reward processing (Sutherland et al., 2012, Luijten et al., 2017). The CEN, anchored in prefrontal and parietal regions, supports inhibitory control, working memory, and goal-directed regulation, whereas the DMN has been consistently associated with self-referential processing and internally oriented cognition (Menon, 2011, Menon, 2023, Molnar-Szakacs and Uddin, 2013, Yeshurun et al., 2021). Contemporary neurobiological models further suggest that addictive behaviors may arise not only from heightened reward-related responding but also from impaired top-down control and disrupted interactions between control-related and self-referential systems. Within this framework, abnormal connectivity in frontal and midline cortical regions may represent an important neural basis of IGD (Menon, 2011, Brand et al., 2019).

Consistent with this view, previous rs-fMRI studies of IGD have documented altered connectivity in networks related to executive control, reward processing, and self-referential or default-mode functions (Ding et al., 2013, Dong et al., 2015, Jin et al., 2016, Zhang et al., 2016, Gao et al., 2025). For instance, Dong et al. reported reduced functional connectivity within the executive control network in IGD, whereas Ding et al. observed altered default network connectivity in adolescents with problematic gaming behavior (Ding et al., 2013, Dong et al., 2015). More generally, reviews and recent network-level neuroimaging studies suggest that IGD is associated with abnormalities spanning control-, reward-, and motivation-related systems, although the precise loci and direction of these effects differ across studies (Fauth-Bühler and Mann, 2017, Weinstein and Lejoyeux, 2020, Yao et al., 2017, Gao et al., 2025).

However, results across individual RSFC studies have remained heterogeneous, probably because of differences in sample characteristics, seed selection, imaging pipelines, and analytic strategies. This variability has made it difficult to determine which neural abnormalities in IGD are robust and reproducible. Coordinate-based synthesis offers one way to address this issue by integrating spatially distributed neuroimaging findings across studies and identifying areas of convergence (Müller et al., 2018, Radua et al., 2012, Taylor et al., 2021). Even so, convergence in a meta-analysis does not by itself demonstrate reproducibility in an independent sample. For this reason, using key meta-analytically identified clusters as targets for focused validation in a separate cohort provides a practical strategy for testing whether the most stable abnormalities can be replicated within a unified analytic framework (Yan et al., 2021, Niu et al., 2022).

Accordingly, the present study adopted a two-stage design. First, we carried out an AES-SDM analysis of resting-state functional connectivity studies involving IGD-related conditions to identify spatially convergent abnormalities. Second, clusters derived from the meta-analysis in the middle frontal gyrus and a midline medial frontal/cingulate-related region were used as seeds for independent resting-state functional connectivity analyses in an IGD group and matched healthy controls. Guided by the I-PACE framework and large-scale network models of psychopathology, we hypothesized that IGD would exhibit reproducible connectivity abnormalities involving frontal executive-control-related regions and medial self-referential regions, and that these alterations would be related to symptom severity.

## 2 Methods

### 2.1 Study 1: Anisotropic Effect Size–Signed Differential Mapping (AES-SDM) Meta-Analysis

#### 2.1.1 Study Registration and Guideline

This meta-analysis was conducted in accordance with the Preferred Reporting Items for Systematic Reviews and Meta-Analyses (PRISMA) guidelines (Page et al., 2021). The study protocol was prospectively registered in the International Prospective Register of Systematic Reviews (PROSPERO) (Registration ID: CRD420251207170).

#### 2.1.2 Literature Search Strategy

A systematic literature search was conducted in PubMed, Web of Science, and Scopus from January 1, 2010 to the date of retrieval. The search strategy combined terms related to internet gaming disorder and closely related internet/gaming-addiction constructs, functional magnetic resonance imaging, and the brain. Detailed search strategies for each database are provided in Supplementary Appendix S1. In addition, the reference lists of relevant meta-analyses and review articles were manually screened to identify further eligible studies. Because broader internet-related addiction terms were used to maximize search sensitivity, studies investigating IGD as well as closely related internet/gaming-addiction conditions were considered for inclusion in Study 1, whereas the independent validation stage focused specifically on IGD.

#### 2.1.3 Inclusion and Exclusion Criteria

Studies were included if they met all of the following criteria: (1) employed resting-state fMRI to investigate functional connectivity; (2) compared individuals with IGD or closely related internet/gaming-addiction conditions and healthy controls (HCs); (3) reported whole-brain analyses and/or a priori defined regions of interest (ROIs); (4) provided peak coordinates in Montreal Neurological Institute (MNI) or Talairach space; and (5) used standardized diagnostic criteria (e.g., DSM) and/or validated assessment instruments (e.g., IAT, YDQ, or DICA) to define IGD-related conditions. Studies were excluded if they (1) did not use fMRI, (2) failed to report peak coordinates and such data could not be obtained from the authors, or (3) did not include a comparison between patients and healthy controls. When a study divided participants into multiple subgroups, the subgroup without comorbidities or medication use was preferentially included.

#### 2.1.4 Quality Assessment

The quality of each included study was independently assessed using the Newcastle–Ottawa Scale (NOS). The NOS evaluates three domains for cohort studies: selection, comparability, and outcome, with a maximum of four, two, and three stars, respectively. Accordingly, the highest possible score is nine stars, and studies scoring more than six stars were considered high quality. Any discrepancies were resolved through discussion with a second reviewer and re-evaluation of the original studies.

#### 2.1.5 Data Synthesis and Statistical Analysis

Coordinate-based meta-analysis was performed using anisotropic effect size–signed differential mapping (AES-SDM; version 5.15, https://www.sdmproject.com/), a widely used voxel-wise neuroimaging meta-analytic approach (Radua and Mataix-Cols, 2009, Radua et al., 2012, Radua, 2015, Müller et al., 2018). For each included study, peak coordinates and corresponding statistics (t or z values) representing group differences in functional connectivity between the IGD-related condition group and the HC group were extracted. When necessary, the reported statistics were converted into Hedges’ g effect sizes using the SDM conversion utilities. Individual study maps were recreated by combining peak coordinates and effect sizes under the default anisotropic preprocessing framework of AES-SDM.

A random-effects model was used to generate the mean meta-analytic map while accounting for within-study variance and between-study heterogeneity. In the primary analysis, the isotropic full width at half maximum (FWHM) was set to 20 mm with anisotropy fixed at 1.0, following the default parameter settings for functional MRI/PET in AES-SDM (Radua, 2015, Radua et al., 2012). Statistical significance was defined using the standard AES-SDM thresholds of voxel-level p < 0.005, peak height Z > 1.0, and cluster extent ≥ 10 voxels. Whole-brain jackknife sensitivity analyses were conducted by iteratively removing one study at a time and repeating the meta-analysis to assess the robustness of the main findings. To further evaluate parameter robustness, additional sensitivity analyses were performed using alternative isotropic FWHM values of 15 mm and 25 mm while keeping all other parameters unchanged.

#### 2.1.6 Control for Publication Bias

Potential publication bias was assessed by visual inspection of funnel plots and Egger’s regression test. The corresponding results are presented in Fig. 3.

### 2.2 Study 2: Independent Validation Study with Resting-State fMRI

#### 2.2.1 Participants: Independent Validation Cohort

A total of 58 individuals with Internet Gaming Disorder (IGD) and 38 demographically matched healthy controls (HCs) were recruited for the independent validation study. The diagnosis of IGD was established by two experienced psychiatrists according to the Diagnostic and Statistical Manual of Mental Disorders, Fifth Edition (DSM-5). The severity of internet gaming disorder was assessed using the Young’s Internet Addiction Test (IAT). All participants were right-handed.

The inclusion criteria for the IGD group were as follows: (1) meeting DSM-5 diagnostic criteria for IGD; (2) IAT score ≥ 50; (3) no history of antipsychotic or neuropathic medication use; (4) no recent use of sedative, hypnotic, analgesic, or narcotic drugs; (5) no history of severe craniocerebral trauma or organic brain disease; and (6) no substance abuse or dependence other than IGD (e.g., nicotine or alcohol). The inclusion criteria for the HC group were as follows: (1) physical and mental health without any neurological or psychiatric disorder; (2) IAT score < 50; and (3) no history of substance abuse or dependence.

The exclusion criteria for all participants were as follows: (1) intellectual disability; (2) contraindications to MRI, such as metal implants, pacemakers, or claustrophobia; (3) pregnancy or lactation; and (4) current or previous psychiatric disorders or hereditary neurological disorders. In the IGD group, symptom severity was further assessed using the Hamilton Anxiety Scale (HAMA) and the Hamilton Depression Scale (HAMD). All participants or their guardians were informed of the study purpose, procedures, contraindications, and possible discomforts before the examination, and written informed consent was obtained. The study was approved by the Ethics Committee of the First Affiliated Hospital of Zhengzhou University and was conducted in accordance with the Declaration of Helsinki.

#### 2.2.2 MRI Data Acquisition

MRI data were acquired on a 3.0-T Siemens Prisma scanner (Siemens Healthcare, Erlangen, Germany) equipped with a 64-channel head coil. During scanning, foam pads and earplugs were used to minimize head motion and reduce scanner noise. Participants were instructed to keep their eyes closed, remain awake, stay still, and avoid falling asleep during the scan. After scanning, all participants reported that they had remained awake throughout the acquisition.

High-resolution T1-weighted structural images were acquired using a 3D magnetization-prepared rapid gradient echo sequence with the following parameters: repetition time (TR) = 2300 ms; echo time (TE) = 2.32 ms; flip angle = 9°; matrix size = 256 × 256; slice thickness = 0.9 mm; slice gap = 0 mm; number of slices = 176; voxel size = 0.9 × 0.9 × 0.9 mm^3^; and field of view (FOV) = 240 × 240 mm^2^.

Resting-state functional images were acquired using a gradient-echo echo-planar imaging sequence with the following parameters: TR = 1000 ms; TE = 30 ms; flip angle = 70°; matrix size = 64 × 64; slice thickness = 2.2 mm; slice gap = 0.5 mm; number of slices = 52; voxel size = 2 × 2 × 2.2 mm^3^; FOV = 220 × 220 mm^2^; and 400 whole-brain volumes.

#### 2.2.3 fMRI Data Preprocessing

Resting-state fMRI data were preprocessed using the advanced edition of the Data Processing Assistant for Resting-State fMRI (DPARSFA, version 5.0; https://rfmri.org/DPABI) implemented in MATLAB (Yan et al., 2016, Yan and Zang, 2010). The preprocessing steps were as follows: (1) DICOM images were converted to NIfTI format; (2) the first 10 volumes were discarded to allow for signal stabilization; (3) slice timing correction was performed; (4) head motion correction was conducted, and participants with maximum translation > 2 mm or rotation > 2° were excluded; (5) functional images were spatially normalized to the standard EPI template and resampled to 3 × 3 × 3 mm^3^; (6) linear trends were removed; (7) temporal band-pass filtering (0.01–0.08 Hz) was applied; and (8) nuisance covariates, including the Friston 24 head-motion parameters, white matter signals, and cerebrospinal fluid signals, were regressed out (Friston et al., 1996). Global signal regression was not performed. To further minimize motion-related effects, volumes with framewise displacement (FD) > 0.2 mm were identified and censored using a spline interpolation approach (Power et al., 2012, Yan et al., 2016).

#### 2.2.4 Seed-Based Functional Connectivity and Statistical Analysis

Based on the consistent clusters identified in the meta-analysis (Study 1), peak coordinates within the middle frontal gyrus and midline medial frontal/cingulate-related region were defined as regions of interest (ROIs). Spherical ROIs (radius = 5 mm) were generated centered on these coordinates. Before seed-based functional connectivity calculation, the normalized functional images were spatially smoothed in MATLAB using an 8 mm full width at half maximum (FWHM) Gaussian kernel.

For each participant, the mean time series of each ROI was extracted from the smoothed functional images. Voxel-wise Pearson correlation coefficients were then calculated between the ROI time series and the time series of all other brain voxels to generate individual whole-brain functional connectivity maps. The resulting correlation maps were transformed into z values using Fisher’s r-to-z transformation to improve normality.

Group differences in seed-based functional connectivity were examined using two-sample t-tests implemented in Statistical Parametric Mapping (SPM12, http://www.fil.ion.ucl.ac.uk/spm/). Statistical significance was determined using Gaussian Random Field (GRF) correction with a voxel-level threshold of p < 0.005 and a cluster-level threshold of p < 0.05. Demographic and clinical characteristics were compared between groups using independent-samples t-tests implemented in SPSS (version 26, IBM Corp., Armonk, NY, USA), with the significance level set at p < 0.05 (two-tailed). In addition, Pearson correlation analyses were conducted to examine associations between altered seed-based functional connectivity values and clinical measures within the IGD group. False discovery rate (FDR) correction was applied to control for multiple comparisons.

## 3 Results

### 3.1 Results of the meta-analysis

#### 3.1.1 Study Selection and Characteristics of Included Studies

**Fig. 1:**
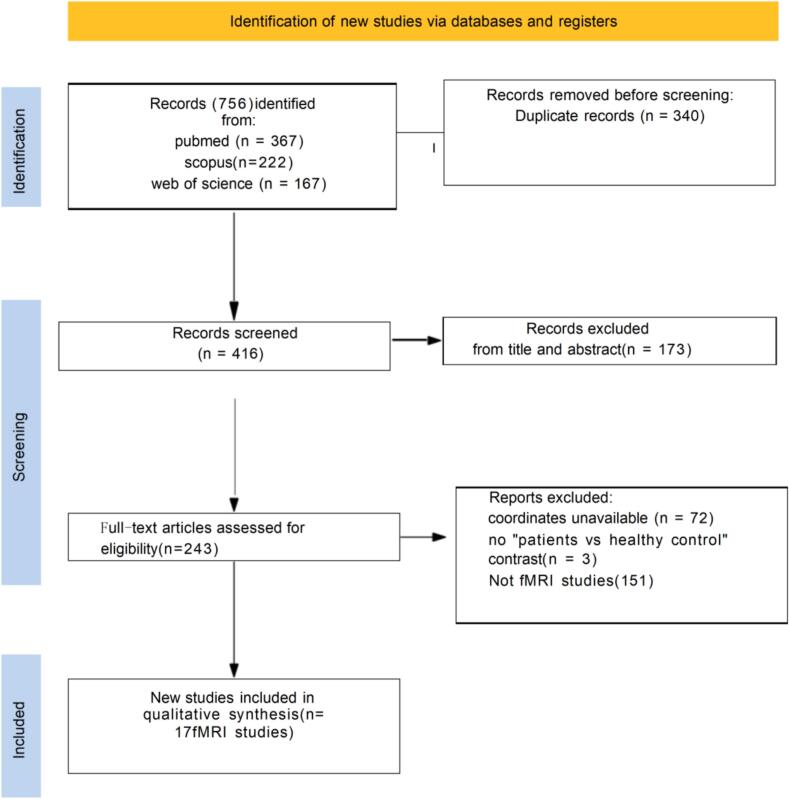
PRISMA flow diagram of study selection for the meta-analysis. fMRI, functional magnetic resonance imaging; DSM, Diagnostic and Statistical Manual of Mental Disorders; IAD, Internet addiction disorder; IAT, Internet Addiction Test; ICD, International Classification of Diseases; IGD, Internet Gaming Disorder; NA, not available.

As shown in Fig. 1, 756 records were identified through database searching. After removal of 340 duplicates, 416 records remained for title and abstract screening. Following exclusion of 173 records, 243 full-text articles were assessed for eligibility. Of these, 226 were excluded due to unavailable peak coordinates (n = 72), absence of a patient–control comparison (n = 3), or not using fMRI (n = 151). Ultimately, 17 resting-state fMRI studies met the inclusion criteria and were included in the meta-analysis. These studies encompassed IGD as well as closely related internet/gaming-addiction conditions (e.g., IA, IAD, and IGA), whereas the independent validation cohort in Study 2 consisted specifically of individuals with DSM-5-defined IGD. Across the 17 included studies, the meta-analysis encompassed 544 individuals with IGD-related conditions and 615 healthy controls (total N = 1,159).

#### 3.1.2 Quality assessment

The 17 included rs-fMRI studies had a mean NOS score of 6.82, and all were rated as high quality (NOS score ≥ 6). Detailed scores are presented in Table 1.

**Table 1:** NOS scores of the 17 included rs-fMRI studies

Study	Selection				Comparability	Outcome			Scores
	Representativeness of the exposed cohort	Selection of the non-exposed cohort	Ascertainment of exposure	Demonstration that outcome of interest was not present at start of study	Control for important factors*	Assessment of outcome	Was follow-up long enough for outcomes to occur	Adequacy of follow-up of cohorts	
Ding et al. (2013)	★	★	★	★	★★	★			7
Dong et al. (2015)	★	★	★	★	★★	★			7
Gao et al. (2015)	★	★	★	★	★★	★			7
Lin et al. (2015)	★	★	★	★	★★	★			7
Wang et al. (2015)	★	★	★	★	★★	★			7
Jin et al. (2016)	★	★	★	★	★★	★			7
Zhang et al. (2016)	★	★	★	★	★★	★			7
Du et al. (2017)	★	★	★	★	★★	★			7
Xu et al. (2018)	★		★	★	★★	★			6
Seok et al. (2018)	★		★	★	★★	★			6
Kim et al. (2019)	★		★	★	★★	★			6
Wang et al. (2019)	★	★	★	★	★★	★			7
Cheng et al. (2020)	★	★	★	★	★★	★			7
Dong et al. (2021)	★		★	★	★★	★			6
Kim et al. (2021)	★	★	★	★	★★	★			7
Lee et al. (2021)	★		★	★	★★	★	★	★	8
Quan et al. (2022)	★	★	★	★	★★	★			7

Note. The Newcastle–Ottawa Scale (NOS) evaluates study quality across three domains: selection (maximum 4 stars), comparability (maximum 2 stars), and outcome (maximum 3 stars). Studies with NOS scores ≥ 6 were considered high quality. * Comparability was scored for control of important confounding factors.

#### 3.1.3 Meta-analysis identified functional connectivity alterations in cognitive control–related regions across IGD-related conditions

As shown in ​Table 2​, the demographic and clinical characteristics of the included resting-state fMRI studies are summarized. The main meta-analytic results arepresented in ​Table 3​.

**Table 2:** Demographic characteristics of included rs-fMRI studies in IGD-related conditions

Study	IGD-related n	Sex (M/F)	Age (years)	HC n	Sex (M/F)	Age (years)	Diagnosis	Clinical measure	Task
Ding et al. (2013)	17	13/4	16.94	24	16/8	15.87	IGA	YDQ	NA
Dong et al. (2015)	35	35/0	22.21	36	36/0	22.81	IGD	IAT	Stroop
Gao et al. (2015)	30	30/0	23.57	30	30/0	24.23	IGD	DICA	NA
Lin et al. (2015)	14	12/2	17.12	15	13/2	17.87	IAD	NA	NA
Wang et al. (2015)	17	13/4	16.94	24	18/6	15.87	IGD	DSM-5	NA
Jin et al. (2016)	25	16/9	19.12	21	14/7	18.76	IGD	DSM-5	NA
Zhang et al. (2016)	19	17/2	20.80	19	17/2	21.50	IGD	IAT	NA
Du et al. (2017)	27	27/0	17.07	35	35/0	16.80	IGD	IAT	NA
Xu et al. (2018)	30	13/17	20.83	30	12/18	21.20	IGD	YDQ	NA
Seok et al. (2018)	20	20/0	21.70	20	20/0	22.40	IGD	IAT	NA
Kim et al. (2019)	22	NA	28.27	24	NA	28.17	IGD	DSM-5	NA
Wang et al. (2019)	28	21/7	21.32	30	22/8	21.73	IA	YDQ/IAT	NA
Cheng et al. (2020)	28	22/6	20.70	24	18/6	21.00	IA	YDQ	NA
Dong et al. (2021)	130	79/51	21.98	207	131/76	21.51	IGD	IAT/DSM-5	NA
Kim et al. (2021)	50	NA	23.30	22	NA	23.50	IGD	DSM-5	NA
Lee et al. (2021)	22	22/0	23.90	19	19/0	23.90	IGD	DSM-5	NA
Quan et al. (2022)	30	30/0	30.30	35	35/0	35.00	IGD	DSM-5	NA

Note. Age values are reported as study means, as extracted from the original articles. IGD-related conditions in Study 1 included IGD as well as closely related internet/gaming-addiction constructs (e.g., IA, IAD, and IGA). IGD = Internet Gaming Disorder; IA = Internet Addiction; IAD = Internet Addiction Disorder; IGA = Internet Gaming Addiction; HC = healthy control; IAT = Internet Addiction Test; YDQ = Young’s Diagnostic Questionnaire; DICA = Diagnostic Interview for Children and Adolescents; DSM-5 = Diagnostic and Statistical Manual of Mental Disorders, Fifth Edition; NA = not available.

**Table 3:** Convergent RSFC abnormalities identified by AES-SDM meta-analysis in IGD-related conditions

Increased connectivity				
Region	Peak MNI coordinate (x, y, z)	Cluster size (voxels)	SDM-Z	p value
Median cingulate/paracingulate region	-4, -10, 36	2078	2.884	< 0.001
Right middle frontal gyrus, BA 46	28, 44, 32	418	1.873	0.0005
Left middle frontal gyrus, BA 45	-44, 38, 26	314	1.783	0.0010
Decreased connectivity				
Region	Peak MNI coordinate (x, y, z)	Cluster size (voxels)	SDM-Z	p value
Left inferior frontal gyrus, orbital part, BA 47	-46, 22, -8	383	-2.613	0.0002
Midline medial frontal/cingulate-related region (cluster 1)	-10, 54, 22	28	-2.106	0.0020
Midline medial frontal/cingulate-related region (cluster 2)	14, -16, 62	16	-2.159	0.0016
Right arcuate network, posterior segment	50, -40, -2	20	-2.149	0.0016

Note. This table summarizes convergent coordinate-based RSFC abnormalities identified by AES-SDM rather than pairwise FC edges. Anatomical labels in the main “Region” column were harmonized for consistency across the manuscript. Statistical significance was defined as voxel-level p < 0.005, peak height Z > 1.0, and cluster extent ≥ 10 voxels. All identified clusters were preserved across leave-one-out jackknife analyses.

**Fig. 2:**
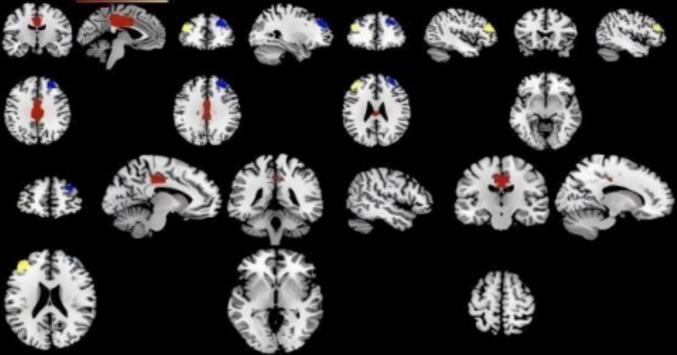
Brain regions showing significant functional connectivity alterations in individuals with IGD-related conditions compared with healthy controls based on the AES-SDM meta-analysis. Regions with increased functional connectivity are shown in red and yellow, whereas regions with decreased functional connectivity are shown in blue. Activation clusters are displayed in sagittal, coronal, and axial views. Statistical significance was set at voxel-level p < 0.005, peak height Z > 1.0, and cluster extent ≥ 10 voxels. Abbreviations: IGD, Internet Gaming Disorder; HCs, healthy controls; MNI, Montreal Neurological Institute; SDM, signed differential mapping.

As illustrated in Fig. 2, compared with healthy controls, individuals with IGD-related conditions showed significantly enhanced functional connectivity in the median cingulate/paracingulate region (MNI [−4, −10, 36]), the right middle frontal gyrus (BA 46; MNI [28, 44, 32]), and the left middle frontal gyrus (BA 45; MNI [−44, 38, 26]). In contrast, significantly weakened functional connectivity was observed in the left orbitofrontal cortex (BA 47; MNI [–46, 22, –8]), the midline medial frontal/cingulate-related region (MNI [–10, 54, 22] and [14, –16, 62]), and the right posterior arcuate fasciculus (MNI [50, –40, –2]).

#### 3.1.4 Sensitivity analysis across different FWHM settings

To further assess parameter robustness, additional AES-SDM analyses were performed using alternative isotropic FWHM values of 15 mm and 25 mm while keeping all other parameters unchanged. The principal findings remained broadly consistent across all three analyses. Specifically, the major positive clusters in the median cingulate/paracingulate region and bilateral middle frontal gyrus were preserved across settings. Likewise, the major negative clusters in the left orbital inferior frontal gyrus, midline medial frontal/cingulate-related clusters, and the posterior network-related region were also retained. Although minor variations in peak coordinates, cluster extent, and SDM-Z values were observed, the overall spatial pattern and direction of the findings remained stable. Detailed results are provided in Supplementary Table S2.

#### 3.1.5 Reliability Analysis and Publication Bias

**Fig. 3:**
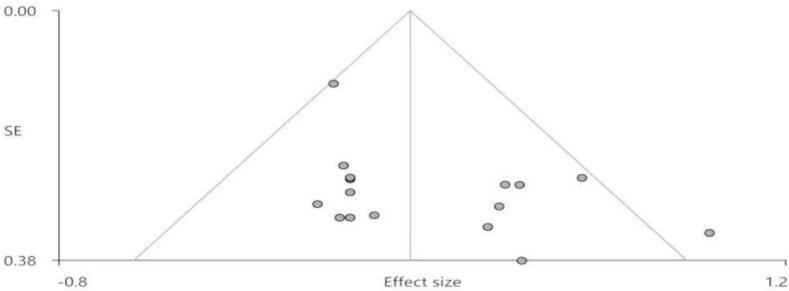
Funnel plot and Egger’s regression test for assessment of publication bias in the meta-analysis.

As shown in Fig. 3, visual inspection of the funnel plot revealed that the majority of studies were distributed within the inverted funnel, showing a relatively balanced distribution of effect sizes across the midline. However, Egger’s regression test indicated a potential, albeit marginal, publication bias (Bias = 1.59, t = 2.21, df = 15, p = 0.043).

To evaluate whether this potential bias influenced the stability of the findings, leave-one-out jackknife sensitivity analyses were performed. The core spatial patterns remained consistent throughout all iterations, demonstrating that the overall results were robust and not disproportionately driven by any single study. Furthermore, the stability of the findings across different FWHM settings confirms the reliability of the identified fronto-midline connectivity abnormalities in IGD.

### 3.2 Results of Seed-Based Functional Connectivity

#### 3.2.1 Demographic and Clinical Characteristics of the Sample

As shown in Table 4, no significant group differences were observed in age (p = 0.183), years of education (p = 0.613), or mean FD (p = 0.380).

**Table 4:** Demographic and clinical characteristics of participants

Variable	IGD (n = 58)	HC (n = 38)	p value
Age (years)	14.79 ± 2.03	15.92 ± 5.90	0.183
Education (years)	8.90 ± 1.87	9.21 ± 4.12	0.613
Mean FD	0.138 ± 0.045	0.130 ± 0.039	0.380
IAT score	63.36 ± 9.51	—	—
HAMD score	22.98 ± 11.60	—	—
HAMA score	15.34 ± 9.95	—	—

Note. Values are presented as mean ± standard deviation. p values were obtained using two-sample t-tests. IAT was used as a screening measure in both groups but is reported here only for the IGD group as a clinical severity indicator. HAMD and HAMA were assessed only in the IGD group to characterize symptom severity and were therefore not available for the HC group.

#### 3.2.2 Independent validation revealed increased connectivity

**Fig. 4:**
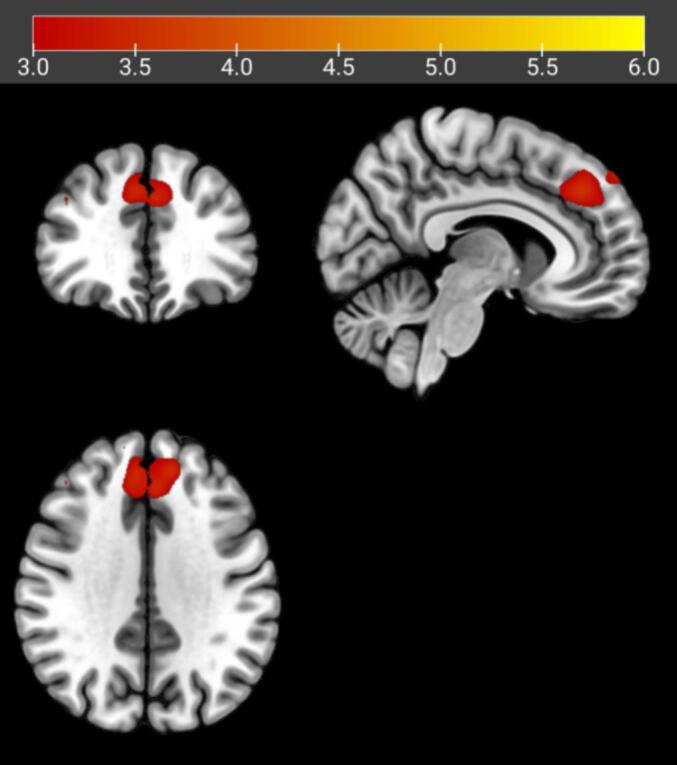
Brain regions showing significantly increased resting-state functional connectivity (RSFC) with the middle frontal gyrus seed in the Internet Gaming Disorder (IGD) group compared with healthy controls. Results are displayed in axial, coronal, and sagittal views. Warm colors indicate increased RSFC. Threshold: voxel-level P < 0.005, cluster-level P < 0.05 (GRF corrected). Color bar represents t values.

**Fig. 5:**
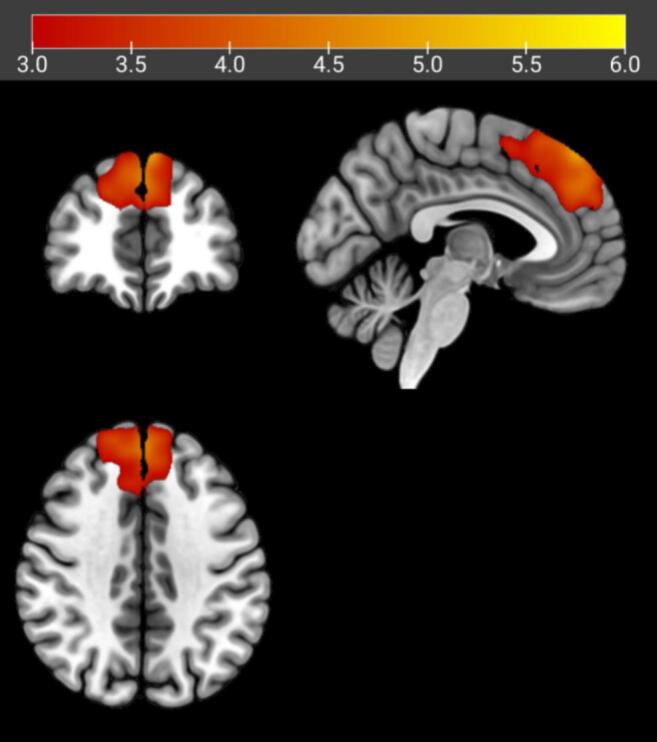
Brain regions showing significantly increased resting-state functional connectivity (RSFC) with the midline medial frontal/cingulate-related seed in the Internet Gaming Disorder (IGD) group compared with healthy controls. Results are displayed in axial, coronal, and sagittal views. Warm colors indicate increased RSFC. Threshold: voxel-level P < 0.005, cluster-level P < 0.05 (GRF corrected). Color bar represents t values.

As shown in Table 5 and Fig. 4, Fig. 5, seed-based functional connectivity analyses revealed significant hyperconnectivity in the IGD group for both the middle frontal gyrus seed and the midline medial frontal/cingulate-related seed.

**Table 5:** Intergroup differences in seed-based functional connectivity in the independent validation cohort

Seed region	Peak region	Peak MNI coordinate (x, y, z)	Cluster size (voxels)	t value
Middle frontal gyrus seed	Frontal_Sup_Medial_L	-3, 32, 40	173	3.22
	Frontal_Sup_Medial_R	6, 40, 40	167	3.64
	Frontal_Sup_R	-12, 44, 40	98	3.03
	Frontal_Sup_L	14, 55, 40	116	2.93
Midline medial frontal/cingulate-related seed	Frontal_Sup_Medial_L	-1, 47, 46	409	4.49
	Frontal_Sup_Medial_R	8, 55, 46	353	4.34
	Frontal_Sup_R	15, 52, 49	288	3.85
	Frontal_Sup_L	-13, 48, 46	247	3.32

Note. Marked regions indicate brain areas showing significantly increased functional connectivity with the middle frontal gyrus seed or the midline medial frontal/cingulate-related seed in the IGD group compared with healthy controls. Results were thresholded using GRF correction at voxel-level p < 0.005 and cluster-level p < 0.05. MNI = Montreal Neurological Institute.

Specifically, using the midline medial frontal/cingulate-related seed, compared with healthy controls, individuals with IGD exhibited significantly increased resting-state functional connectivity (RSFC) with multiple medial and superior frontal regions, including the left superior medial frontal gyrus (Frontal_Sup_Medial_L), right superior medial frontal gyrus (Frontal_Sup_Medial_R), left superior frontal gyrus (Frontal_Sup_L), and right superior frontal gyrus (Frontal_Sup_R) (GRF corrected, voxel-level P < 0.005, cluster-level P < 0.05).

Similarly, the middle frontal gyrus seed showed significantly increased RSFC with the Frontal_Sup_Medial_L, Frontal_Sup_Medial_R, Frontal_Sup_L, and Frontal_Sup_R regions in the IGD group relative to healthy controls (GRF corrected, voxel-level P < 0.005, cluster-level P < 0.05).

#### 3.2.3 Analysis of the Correlation Between Resting-State Functional Connectivity and Clinical Scales

**Fig. 6:**
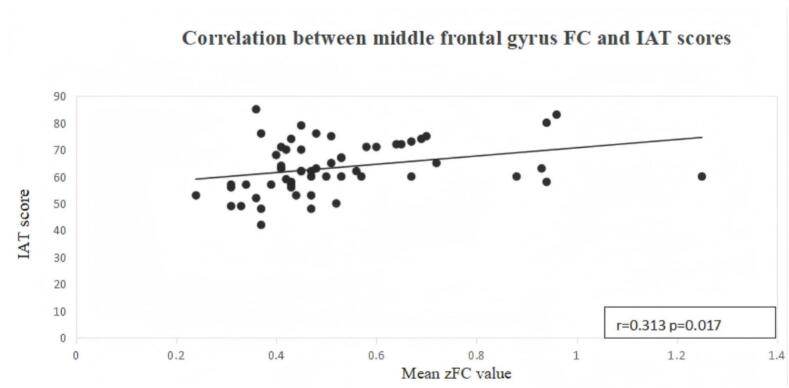
Scatter plot showing the relationship between altered seed-based functional connectivity (zFC values) in the largest significant cluster associated with the middle frontal gyrus seed and Internet Addiction Test (IAT) scores in the IGD group (n = 58). Each dot represents one participant. The solid line indicates the linear regression fit. Pearson correlation analysis revealed a significant positive correlation (r = 0.313, uncorrected p = 0.017).

Given that Internet addiction severity represents the core clinical feature of IGD, primary correlation analyses focused on examining the association between altered seed-based functional connectivity and IAT scores. For each seed, mean z-transformed functional connectivity values were extracted from the largest significant cluster showing group differences. Pearson correlation analyses were then performed between these extracted connectivity values and IAT scores within the IGD group. This significant correlation is illustrated in ​Fig. 6​.

In addition, exploratory correlation analyses were conducted between extracted connectivity values and other clinical measures (HAMD, HAMA). False discovery rate (FDR) correction using the Benjamini–Hochberg procedure was applied to control for multiple comparisons in the exploratory analyses. The detailed correlation results are presented in ​Table 6.

**Table 6:** Correlations between altered seed-based functional connectivity and clinical measures in the IGD group

Seed region	Largest cluster (MNI)	Measure	r	p (uncorrected)	p (FDR-corrected)
Middle frontal gyrus seed	(-3, 32, 40)	IAT (primary)	0.313	0.017	—
Middle frontal gyrus seed	(-3, 32, 40)	HAMD (exploratory)	0.205	0.124	>0.05
Middle frontal gyrus seed	(-3, 32, 40)	HAMA (exploratory)	0.126	0.345	>0.05
Midline medial frontal/cingulate-related seed	(-10, 54, 22)	IAT (primary)	0.171	0.198	—
Midline medial frontal/cingulate-related seed	(-10, 54, 22)	HAMD (exploratory)	0.198	0.137	>0.05
Midline medial frontal/cingulate-related seed	(-10, 54, 22)	HAMA (exploratory)	0.178	0.181	>0.05

Note. Pearson correlation analyses were performed within the IGD group. IAT was defined a priori as the primary clinical measure of interest. HAMD and HAMA were included as exploratory clinical measures. False discovery rate (FDR) correction was applied to exploratory correlations. A dash (—) indicates that FDR correction was not applied to primary analyses. Significant correlations are shown in bold.

## 4 Discussion

This study combined a coordinate-based meta-analysis with independent seed-based resting-state functional connectivity (RSFC) validation to investigate reproducible fronto-midline connectivity abnormalities in Internet gaming disorder (IGD). The convergence of spatial patterns across these two analytical approaches suggests that abnormalities within the central executive network (CEN) may constitute an important neural feature of IGD (Brand et al., 2014, Fauth-Bühler and Mann, 2017, Weinstein and Lejoyeux, 2020).

The meta-analysis identified functional connectivity abnormalities in IGD that mainly involved the median cingulate/paracingulate region and the bilateral middle frontal gyrus (MFG). These regions are closely linked to executive control and inhibitory regulation and are broadly consistent with core control-related systems described in previous network models (Seeley et al., 2007, Dosenbach et al., 2006, Cole and Schneider, 2007). More generally, the observed pattern of frontal and midline connectivity abnormalities may reflect disrupted coordination between executive-control-related systems and self-referential processing systems, particularly the default mode network (DMN). This interpretation is in line with previous accounts of IGD-related control dysfunction and altered large-scale network organization (Dong et al., 2015, Wang et al., 2018, Lee et al., 2017, Song et al., 2021, Weinstein and Lejoyeux, 2020, Menon, 2011).

The independent seed-based RSFC analysis further supported the spatial distribution of the altered regions identified in the meta-analysis. Using meta-analytically derived seeds, individuals with IGD showed significantly increased RSFC associated with both the middle frontal gyrus seed and the midline medial frontal/cingulate-related seed, with the main effects located in medial and superior frontal regions relative to healthy controls. Notably, only connectivity strength related to the middle frontal gyrus seed was positively correlated with IAT scores, whereas the midline medial frontal/cingulate-related seed did not show a significant association with IAT. This pattern suggests that functional alterations linked to the middle frontal gyrus may be more directly related to clinically meaningful variation in addiction severity (Dong et al., 2015, Wang et al., 2018, Zhao et al., 2024). One possible explanation is that the MFG may be more directly involved in top-down inhibitory control over gaming-related urges, whereas medial frontal regions may play a broader role in conflict monitoring or self-referential regulation and may therefore not scale as directly with symptom severity (Brand et al., 2014, Menon and D’Esposito, 2022).

From a network perspective, convergent results across the meta-analysis and seed-based validation were observed mainly in frontal and midline regions, suggesting that these areas may represent shared sites of alteration within executive-control-related circuitry in IGD. Rather than reflecting a simple increase or decrease in cognitive capacity, this pattern of altered intra-frontal connectivity may indicate inefficient or dysregulated functional organization within the executive control system. Such an interpretation is consistent with neurocognitive models of addiction that emphasize impaired top-down control and persistent disorder-relevant preoccupation (Brand et al., 2014, Cole and Schneider, 2007, Menon, 2011).

At the same time, the present findings should be interpreted in light of the study design. Egger’s regression test suggested potential publication bias, indicating that small-study effects or selective reporting cannot be fully excluded. Even so, the core spatial patterns remained highly stable across jackknife sensitivity analyses and across alternative FWHM settings (15 mm and 25 mm), suggesting that the principal findings were robust despite these limitations (Müller et al., 2018, Radua et al., 2012, Eickhoff et al., 2016, Shah et al., 2016, Li et al., 2023).

Taken together, the meta-analytic and validation findings indicate that IGD is characterized by altered functional integration in frontal and midline systems involved in executive control and self-referential processing. The reproducible spatial convergence observed in the middle frontal gyrus and medial frontal regions supports their consideration as candidate hubs within the neural framework of IGD. These regions may therefore warrant further attention as potential targets for future longitudinal and intervention-based studies, including neuromodulatory approaches such as transcranial magnetic stimulation (Hanlon et al., 2015). Further work is still needed to determine whether modulation of these nodes is accompanied by measurable improvement in clinical symptoms.

## 5 Limitations

Several limitations should be acknowledged. First, the coordinate-based meta-analysis relied on reported peak coordinates rather than full statistical images. Although AES-SDM is a widely used and validated method, coordinate-based approaches may be less sensitive than image-based meta-analytic techniques to subtle or spatially extended effects. Second, methodological heterogeneity across the included studies, including differences in MRI acquisition, preprocessing procedures, and statistical thresholds, could not be fully controlled and may have contributed to between-study variability. Third, the independent validation cohort was recruited from a single center and was of moderate size, which may restrict the generalizability of the findings. In addition, because the independent validation stage focused on two meta-analytically derived seed regions, the results should be interpreted as a targeted validation of regionally specific abnormalities rather than as an exhaustive assessment of all large-scale networks implicated in IGD. Replication in larger, multi-center samples would strengthen confidence in the reproducibility of the observed abnormalities. Fourth, given the cross-sectional design, no causal inferences can be drawn about the relationship between functional connectivity alterations and IGD. Finally, the present meta-analysis was conducted using the SDM framework available at the time of analysis. Although this approach remains methodologically appropriate, future studies using updated image-based meta-analytic pipelines may further improve robustness and precision.

## 6 Conclusions

In summary, the integration of a coordinate-based meta-analysis with independent resting-state functional connectivity validation yielded convergent evidence for fronto-midline connectivity abnormalities in Internet gaming disorder (IGD). The meta-analysis revealed reproducible functional connectivity alterations that primarily involved the median cingulate/paracingulate region and the bilateral middle frontal gyrus, pointing to abnormal functional integration in regions related to executive control and cognitive regulation. In the independent sample, seed-based analyses further showed altered whole-brain connectivity associated with the middle frontal gyrus seed and the midline medial frontal/cingulate-related seed, with the main effects located in medial and superior frontal regions. Notably, connectivity related to the middle frontal gyrus seed was significantly associated with symptom severity, supporting a measurable brain–behavior relationship in IGD. Overall, the present results indicate that IGD is characterized by altered functional integration in frontal and midline systems involved in executive control and self-referential processing. These regions may merit further investigation as candidate neuroimaging markers and as potential targets for future neuromodulatory interventions, including transcranial magnetic stimulation.

Data availability statement

The original contributions presented in the study are included in the article and its supplementary materials. Further inquiries can be directed to the corresponding author.

## CRediT authorship contribution statement

**Jiawen Tian:** Conceptualization, Data curation, Formal analysis, Investigation, Methodology, Software, Supervision, Validation, Visualization, Writing – original draft, Writing – review & editing. **Hui Zhang:** Supervision. **Xinyu Wang:** Validation. **Hongyu Zhang:** Writing – review & editing. **Longyao Ma:** Supervision. **Bohui Mei:** Validation. **Mengzhe Zhang:** Writing – review & editing. **Yan Lang:** Supervision. **Yarui Wei:** Validation. **Shaoqiang Han:** Supervision. **QingQing Lv:** Validation. **Yong Zhang:** Funding acquisition, Methodology, Project administration, Resources, Writing – review & editing.

## Funding

The authors declare that financial support was received for the research and/or publication of this article. This work was supported by the National Natural Science Foundation of China (Grant No. 82471962), the Scientific Research and Innovation Team of the First Affiliated Hospital of Zhengzhou University (Grant No. QNCXTD2023007), and the Henan Provincial Young and Middle-aged Health Science and Technology Innovation Talent Support Program (Grant No. LJRC2025008).

Ethical approval

The study was approved by the Ethics Committee of the First Affiliated Hospital of Zhengzhou University and was conducted in accordance with the Declaration of Helsinki.

Consent to Participate

All participants or their legal guardians provided written informed consent after being fully informed of the purpose and procedures of the study.

Consent for Publication

The participants provided written informed consent for the publication of their anonymized data.

## Declaration of competing interest

The authors declare that they have no known competing financial interests or personal relationships that could have appeared to influence the work reported in this paper.

## Data Availability

Data will be made available on request.

## Appendix A Supplementary data

Supplementary materials include the detailed literature search strategies (Appendix S1) and the sensitivity analysis results across different FWHM settings (Supplementary Table S2). The following are the Supplementary data to this article:
