# Supplementary material for: Altered Resting-State Functional Connectivity in Internet Gaming Disorder: Convergent Evidence and Independent Validation
Source: Addict Behav Rep. 2026 May 2;23:100703. doi: 10.1016/j.abrep.2026.100703 (PMC13157096; doi:10.1016/j.abrep.2026.100703)
Supplement: Supplementary Data 1 [file mmc1.pdf]

***Supplementary Appendix S1. Detailed search strategies for PubMed, Web of Science, and Scopus***

Studies published from January 1, 2010 onward were retained for screening. For readability, the year filter is displayed explicitly in the Scopus search string.

*PubMed*
*(((("Internet Addiction Disorder"[Mesh]) OR (((((((((((((((((((((((((Addiction Disorder, Internet[Title/Abstract]) OR (Addiction Disorders, Internet[Title/Abstract])) OR (Disorder, Internet Addiction[Title/Abstract])) OR (Disorders, Internet Addiction[Title/Abstract])) OR (Internet Addiction Disorders[Title/Abstract])) OR (Internet Addiction[Title/Abstract])) OR (Addiction, Internet[Title/Abstract])) OR (Addictions, Internet[Title/Abstract])) OR (Internet Addictions[Title/Abstract])) OR (Internet Gaming Disorder[Title/Abstract])) OR (Disorder, Internet Gaming[Title/Abstract])) OR (Disorders, Internet Gaming[Title/Abstract])) OR (Gaming Disorder, Internet[Title/Abstract])) OR (Gaming Disorders, Internet[Title/Abstract])) OR (Internet Gaming Disorders[Title/Abstract])) OR (Smartphone Addiction[Title/Abstract])) OR (Addiction, Smartphone[Title/Abstract])) OR (Addictions, Smartphone[Title/Abstract])) OR (Smartphone Addictions[Title/Abstract])) OR (Social Media Addiction[Title/Abstract])) OR (Addiction, Social Media[Title/Abstract])) OR (Addictions, Social Media[Title/Abstract])) OR (Media Addiction, Social[Title/Abstract])) OR (Media Addictions, Social[Title/Abstract])) OR (Social Media Addictions[Title/Abstract]))) AND ((functional magnetic resonance imaging) OR (fMRI))) AND (brain)*

*Web of Science*
*((TS=(Internet Addiction Disorder OR Addiction Disorder, Internet OR Addiction Disorders, Internet OR Disorder, Internet Addiction OR Disorders, Internet Addiction OR Internet Addiction Disorders OR Internet Addiction OR Addiction, Internet OR Addictions, Internet OR Internet Addictions OR Internet Gaming Disorder OR Disorder, Internet Gaming OR Disorders, Internet Gaming OR Gaming Disorder, Internet OR Gaming Disorders, Internet OR Internet Gaming Disorders OR Smartphone Addiction OR Addiction, Smartphone OR Addictions, Smartphone OR Smartphone Addictions OR Social Media Addiction OR Addiction, Social Media OR Addictions, Social Media OR Media Addiction, Social OR Media Addictions, Social OR Social Media Addictions)) AND TS=(functional magnetic resonance imaging OR fMRI)) AND TS=(brain)*

*Scopus*
*(TITLE-ABS-KEY("brain")) AND (TITLE-ABS-KEY("Internet Addiction Disorder") OR TITLE-ABS-KEY("Internet Gaming Disorder")) AND (TITLE-ABS-KEY("functional magnetic resonance imaging") OR TITLE-ABS-KEY("fMRI")) AND PUBYEAR > 2009 AND PUBYEAR < 2026*
