# Supplementary material for: Altered Resting-State Functional Connectivity in Internet Gaming Disorder: Convergent Evidence and Independent Validation
Source: Addict Behav Rep. 2026 May 2;23:100703. doi: 10.1016/j.abrep.2026.100703 (PMC13157096; doi:10.1016/j.abrep.2026.100703)
Supplement: Supplementary Data 2 [file mmc2.pdf]

Supplementary Table S2. Sensitivity of AES-SDM findings across different isotropic FWHM settings

FWHM	Major positive clusters retained	Representative positive peaks (MNI, SDM-Z, voxels)	Major negative clusters retained	Representative negative peaks (MNI, SDM-Z, voxels)	Overall interpretation
15 mm	Median cingulate /paracingulate region; right middle frontal gyrus; left middle frontal gyrus	(-2, -12, 36), 2.891, 2009; (28, 44, 32), 1.837, 433; (-44, 38, 26), 1.767, 323	Left orbital inferior frontal gyrus; midline medial frontal /cingulate-related clusters (two peaks); posterior network-related region	(-46, 22, -8), -2.546, 370; (12, -16, 62), -2.092, 33; (-10, 54, 22), -2.013, 33; (52, -40, -4), -2.083, 23	Stable
20 mm	Median cingulate /paracingulate region; right middle frontal gyrus; left middle frontal gyrus	(-4, -10, 36), 2.884, 2078; (28, 44, 32), 1.873, 418; (-44, 38, 26), 1.783, 314	Left orbital inferior frontal gyrus; midline medial frontal /cingulate-related clusters (two peaks); posterior network-related region	(-46, 22, -8), -2.613, 383; (-10, 54, 22), -2.106, 28; (14, -16, 62), -2.159, 16; (50, -40, -2), -2.149, 20	Main analysis
25 mm	Median cingulate /paracingulate region; right middle frontal gyrus; left middle frontal gyrus	(0, -10, 38), 2.855, 2116; (28, 44, 32), 1.894, 402; (-44, 38, 26), 1.791, 304	Left orbital inferior frontal gyrus; midline medial frontal /cingulate-related clusters (two peaks); posterior network-related region	(-46, 22, -8), -2.650, 405; (-10, 54, 22), -2.162, 29; (14, -16, 62), -2.198, 14; (52, -42, -4), -2.143, 22	Stable

Table note. All analyses used the same AES-SDM thresholds as the primary analysis: voxel-level $p < 0.005$, peak height $Z > 1.0$, and cluster extent ≥ 10 voxels. Only isotropic FWHM was varied across analyses (15 mm, 20 mm, and 25 mm), whereas all other parameters were kept unchanged. Anatomical labels were harmonized to match the terminology used in the revised manuscript.
